# Competence-based social status and implicit preference modulate the ability to coordinate during a joint grasping task

**DOI:** 10.1038/s41598-021-84280-z

**Published:** 2021-03-05

**Authors:** Sarah Boukarras, Vanessa Era, Salvatore Maria Aglioti, Matteo Candidi

**Affiliations:** 1 Department of Psychology, Sapienza University of Rome, Rome, Italy; 2 Santa Lucia Foundation, Rome, Italy; 3 Sapienza University of Rome and CNLS@Sapienza Istituto Italiano di Tecnologia, Rome, Italy

**Keywords:** Neuroscience, Psychology

## Abstract

Studies indicate that social status influences people’s social perceptions. Less information is available about whether induced social status influences dyadic coordination during motor interactions. To explore this issue, we designed a study in which two confederates obtained high or low competence-based status by playing a game together with the participant, while the participant always occupied the middle position of the hierarchy. Following this status-inducing phase, participants were engaged in a joint grasping task with the high- and low-status confederates in different sessions while behavioural (i.e., interpersonal asynchrony and movement start time) indexes were measured. Participants’ performance in the task (i.e., level of interpersonal asynchrony) when interacting with the low-status partner was modulated by their preference for him. The lower participants’ preference for a low- relative to a high-status confederate, the worse participants’ performance when interacting with the low-status confederate. Our results show that participants’ performance during motor interactions changes according to the social status of the interaction partner.

## Introduction

The ability to coordinate our actions with others’ is fundamental in everyday social situations and requires accurate predictions of the intentions and consequences of partners’ actions^1^ as well as the integration of observed and executed actions^2^. Both processes most likely rely on visuo-motor transformation mechanisms that take place in the human sensorimotor system^3^ throughout the internal, predictive simulation of others’ observed actions^4,5^. One important finding from recent research is that the activity of the human sensorimotor system during action observation is not neutral to the social identity of the observed agent. Studies have shown that the sensorimotor mirroring of observed actions and sensory states is modulated by group membership and skin colour of the perceived partner^6–10^. Moving from passive observation to dynamic motor interactions, previous studies suggest also that the ability to coordinate with another agent in performing a joint grasping action is sensitive to high-level social dimensions such as race^11^, interpersonal perceptions^12^ and the need of others for inferring abstract concepts^13^.

A primary factor in shaping interpersonal relationships is social status, which is defined as “the relative rank of an individual along one or more social dimensions within a given social hierarchy”^14^. Studies have shown that the gaze of high-status individuals induces a stronger cueing effect^15–19^, that high-status individuals are easier to recognize^20^ and they also influence social decision making during economic^21^ and moral^22^ decisions. Performing grasping movements when in the presence of a high-status observer affects movement kinematics^23^. In addition, observing a high-status model facilitates perceptual decisions^24^ and is accompanied by stronger modulations of EEG correlates of face processing (N170 amplitude)^25^ and EEG correlates of social evaluation (P300 amplitude)^26^.

The effects of social status on action simulation have mainly been tested from the point of view of the observer. Participants’ subjective power level^27^ and social status^28^ were found to modulate motor resonance to observed intransitive actions, as indexed by motor evoked potentials (MEP) amplitude and mu suppression, respectively. The social status of the partner also influences action representation in more interactive tasks. For example, action co-representation effects (indexed by stimulus–response compatibility) in a social version of the Simon task (in which each participant has to press a different key in response to a target appearing either in congruent and incongruent spatial locations with respect to the laterality of the response key) are reduced or even absent in participants from a perceived high-status group in Italy (Italians) playing with member of a perceived low-status group in Italy (Albanians) but not in Albanians playing with Italians^29^. These findings suggest that the actions of low-status individuals are co-represented less than those of the high-status ones. However, a series of five experiments conducted by Farmer and colleagues found no effect of status nor power on the automatic imitation of task-irrelevant finger movements performed by high versus low status virtual avatars or human models^30^. As pointed out by the authors of those experiments, it is possible that social status only affects motor responses to naturalistic, whole-hand actions which require the observer to put specific attention on the model’s movements.

The Joint Grasping Task that we developed^12,31–34^ offers the opportunity to measure different aspects of online motor interactions, from interpersonal synchrony to the fine-grained kinematics of individual grasping movements. In this task, pairs of participants are requested to coordinate their movements to synchronously grasp two bottle-shaped objects either in the upper part (via a Precision grasping) or in the lower part (via a Power grasping). The analysis of interpersonal synchrony performance allows an estimation of the extent to which the two interactors are able to predict the movements of their partner and to integrate each other’s motor plan into their own. The analysis of motion kinematics allows for the isolation of ‘fingerprints’ of signalling strategies^33^ and visuo-motor interference effects during complementary actions^33–39^ bridging the effects described by sensorimotor communication^40^ and automatic-imitative behaviour^41^ theories.

Since social status has a deep influence on the way we process other people and how we simulate their actions, we hypothesised that the social status of the interactor might also modulate the *ability* to coordinate in performing a joint action. In the present study we tested this hypothesis, capitalising on the results of our previous study^42^, in which we demonstrated that perceived competence-based social status of individuals acquired in the context of an interactive game modulates implicit preference for them. Here, participants underwent a status-inducing procedure similar to that used in our previous study^42^ before they were asked to interact with high- and low-status partners and synchronise their reach-to-grasp movements with them. Changes in implicit preference for the high- and low- status partner after the status-inducing procedure were measured by means of the Affect Misattribution Procedure (AMP)^43^. Our results show that the interaction between the perceived social status of the partner and the implicit preference for him modulates participants’ ability to coordinate during dyadic motor interactions.

## Materials and methods

### Participants

Twenty-four right-handed male subjects (age = 24.3 ± 4.2 years) were recruited from the Sapienza University campus. All participants had normal or corrected-to-normal vision, were free from any psychiatric or neurological disorders and were naïve as to the real purpose of the experiment. In order to involve only same-gender dyads, we only tested male participants as the two confederates were males. Five participants were excluded from the final sample as they expressed suspicion about the cover story (final sample comprised 19 male subjects, age = 23.8 ± 4.1 years). The sample size was indicated as adequate by a power analysis performed with the software More Power 6.0.4^44^ for obtaining a power of 0.90 given an alpha level of 0.05 and an expected effect size of partial eta squared = 0.4 in a 2 × 2 × 2 × 2 within factors design. The expected effect size was selected from a previous study that used the same task to assess the influence of the interactor’s social identity on dyadic interaction^11^. Participants gave their written informed consent to participate in the study and received a reimbursement of 14 euros. The experimental protocol was approved by the ethics committee of the IRCCS Fondazione Santa Lucia (Rome, Italy) and was carried out in accordance with the ethical standards of the 1964 Declaration of Helsinki. Informed consent was obtained for publication of identifying images in an online open-access publication.

### Implicit evaluation task (Affect Misattribution Procedure)

Participants were engaged in a modified version of the AMP. Nineteen Chinese characters (on a grey background, 512 × 384 pixels) were used as target stimuli (i.e., those that were evaluated by participants). Prime stimuli were two pictures (292 × 400 pixels) of the two male confederates’ face (Confederate 1 and Confederate 2) whereas the backward masks were two same-size scrambled versions of the original pictures of the confederates’ faces, created with Matlab (Mathworks, Cherborn, MA, USA), see Supplementary Materials for details. Participants were informed that they would see one image (no mention was given concerning the fact that this would show the identity of the confederates) followed by a Chinese pictograph, and that they should ignore the first image and rate the pleasantness of the second stimulus by clicking with the mouse on the VAS point that corresponded to their judgment. The rationale for the AMP is that the (positive or negative) affect elicited by the prime picture is transferred to the neutral target. It follows that target pictures preceded by positive images are evaluated as more pleasant than those preceded by negative primes.

Throughout two blocks of 19 trials, each target image was repeated twice, once preceded by the face of Confederate 1 and once by the face of Confederate 2 as primes. This procedure was performed three times: (1) soon after meeting the partners to measure individuals’ baseline preference; (2) after the status-inducing procedure (see below), to measure the effect of the status-induction; and (3) after the joint action task (see below), to measure if interacting with the confederates had changed the preference for high-/low-status partners compared to the pre-interaction condition.

### Status-inducing procedure

**Figure 1:**
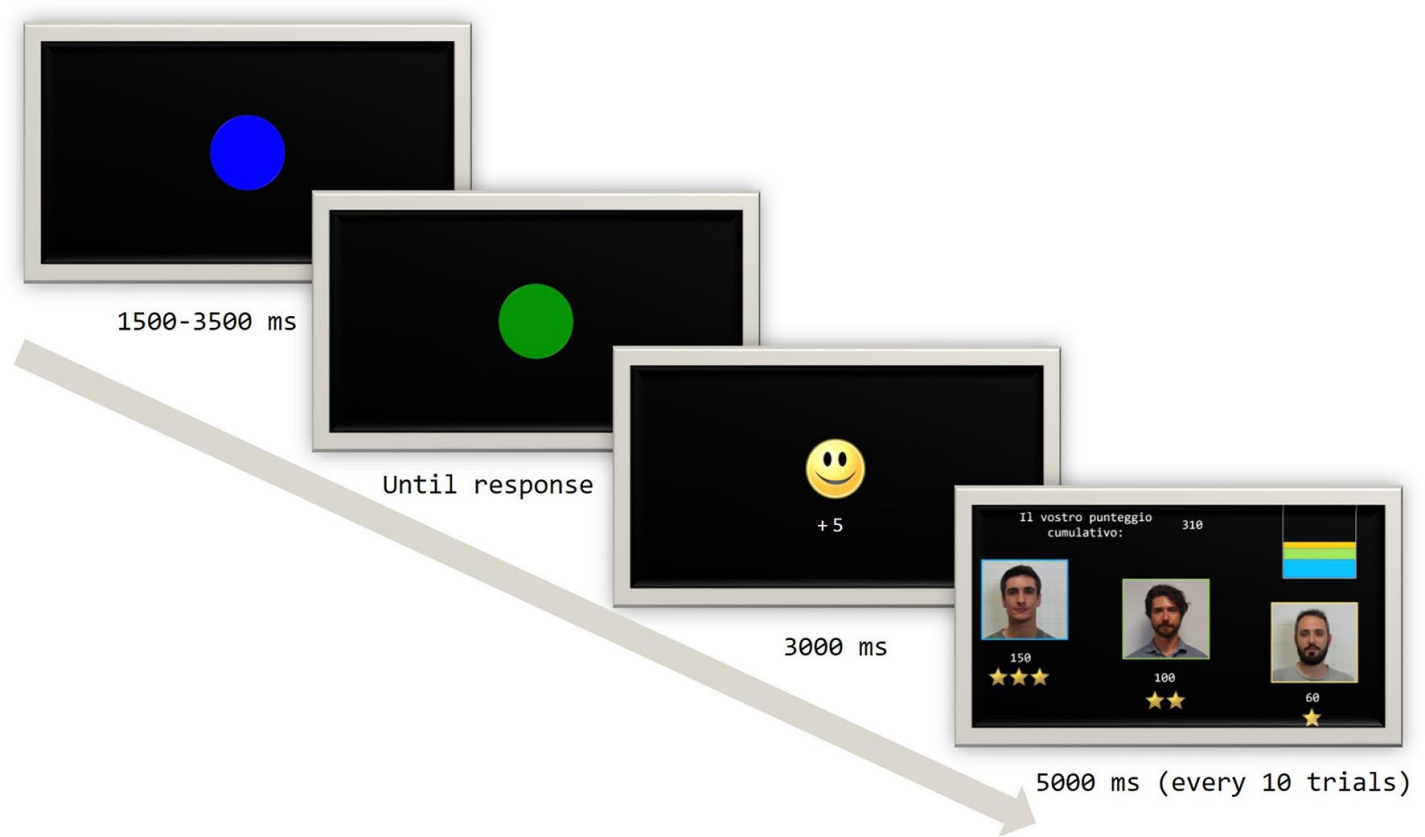
Status-inducing procedure.

Subjects were engaged in a cooperative time estimation task with the two confederates, adapted from Boksem et al.^45^, see Supplementary Information for a detailed description. Importantly, they were told that the individual score of each player would contribute to a collective score and that at the end of the experiment this score would be converted in real money to be split equally between the three players. At the start of each trial, a blue circle appeared on the computer screen for a random time interval (1500–3500 ms), after which it turned green. Participants were asked to press the space bar exactly 1 s after the colour changed. Participants were told that the other two individuals (i.e., the confederates) were performing the same task in two separate rooms. Participants received a feedback after each trial (a smiley face for correct responses and a sad face for errors) and could see their individual cumulative score. Every 10 trials a collective feedback slide was presented, displaying the pictures of the three players, each one framed with a distinct colour. Below each picture, the individual score and a different number of stars (3 stars for the best player, 2 for the middle and 1 for the worst) were displayed. To further highlight the differential contribution of each player in the game, a square containing horizontal bars in different colours (the same as the pictures frames) that had a different width according to respective player’s contribution was presented (Fig. 1). At the end of the procedure participants were asked to rate the two confederates in terms of Competence, Intelligence, Dominance and Attractiveness on a 10-points scale. Each question (e.g. “How much do you think this person is competent?”) was presented on E-prime and participants answered by pressing the correspondent number on the keyboard. The status attribution for the two confederates was counterbalanced across subjects, so that for 8 subjects Confederate 1 was the high status (i.e. the best player) and Confederate 2 was the low status (i.e. the worst player), while the attribution was reversed for the remaining 11 subjects.

### Joint grasping task

**Figure 2:**
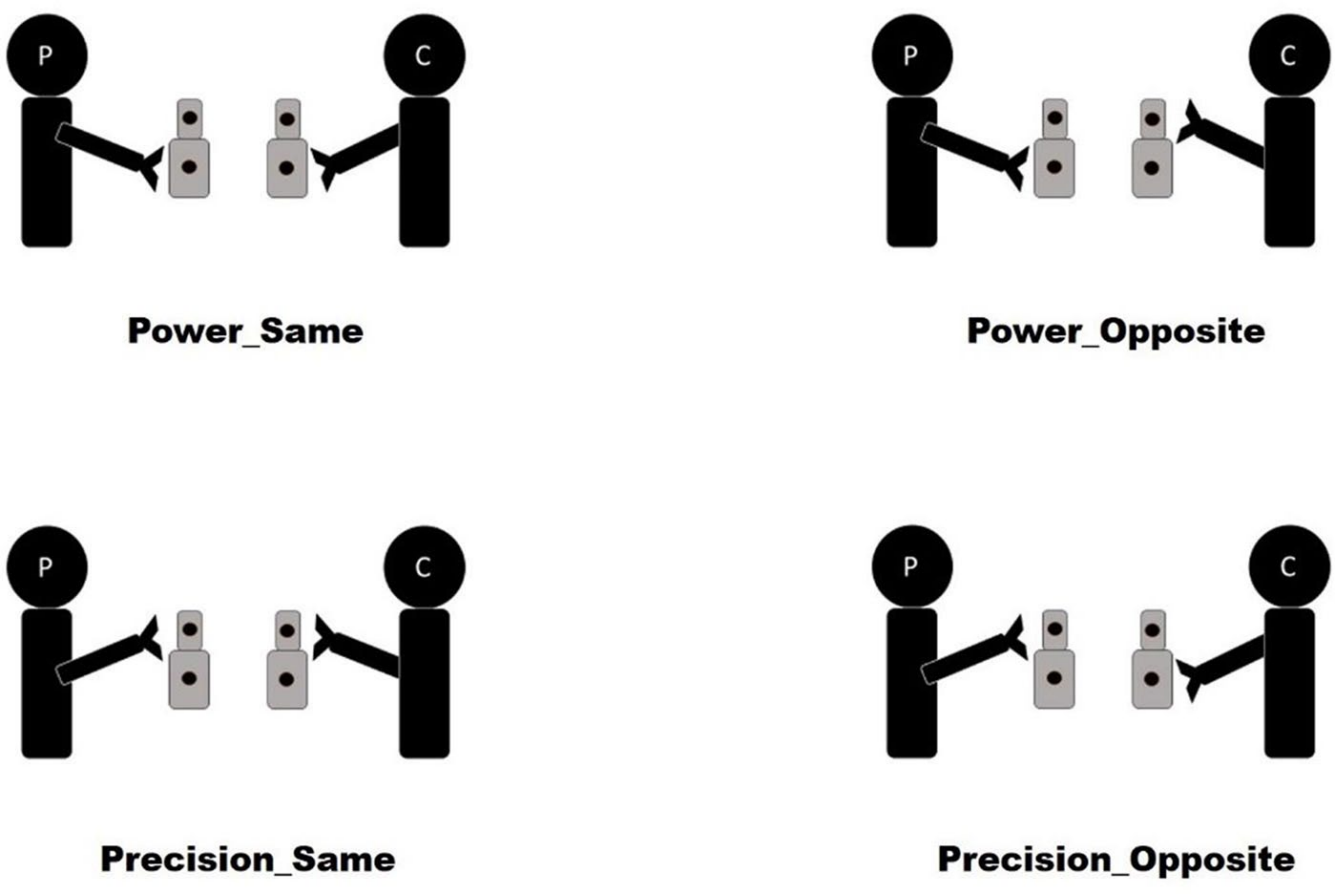
Graphical representation of the Joint Grasping task. Power and Precision trials are considered from the point of view of the Participant (P, on the left side). Opposite and Same movements are considered from the point of view of both the Participant and the Confederate (C, on the right side).

Participants were then asked to perform a Joint-Grasping Task^31–35^ (Fig. 2, see Supplementary Information for a detailed description of the task). Participants were asked to synchronize their reach-to-grasp movements with those of the interaction partner in two different tasks. In the “Interactive” task, participants were instructed to perform an imitative (“same”) or complementary (“opposite”) movement (i.e., if the instruction was to perform a complementary movement, one participant grasps the lower part of the object and the partner grasps the upper part, or vice versa). In the “Cued” task, participants were instructed to grasp the lower part of the object with a power grip after hearing a “low pitch” sound and the upper part with a precision grip after hearing a “high pitch” sound. In both tasks, the two participants received the auditory instruction at the same time. This means that any difference between the two start times (i.e., Start Delay, see below) cannot be attributed to a delay in receiving the instruction. Moreover, in the Interactive task the two participants received the very same instruction (i.e. “opposite” or “same”), meaning that participants did not know in advance what action to perform (i.e. power or precision grip) or which of the two would start first, but they needed to coordinate in real-time. The two tasks are equivalent in terms of the individual movements to be performed. However, in the Interactive task participants were required to predict other’s actions and to mutually adapt in order to successfully interact. In the Cued task, instead, participants were already instructed about what action to perform and needed only to coordinate their timing in order to grasp the objects synchronously with the partner. Subjects completed one Interactive and one Cued block with each confederate (high status and low status, in across-participants counterbalanced order). In both tasks, participants performed four types of grasping-interaction trials: Power-Same, Power-Opposite, Precision-Same, Precision-Opposite (see Fig. 2; power and precision labels refer to the movement executed by the participant). The Interactive block comprised 60 trials (30 Imitative and 30 Complementary). The Cued Block comprised 60 trials (30 Precision and 30 Power for both the participant and the confederate, the combination resulting in 30 Imitative and 30 Complementary).

### General procedure

**Figure 3:**
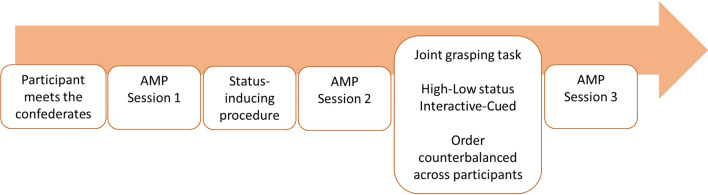
Experiment timeline. AMP = Affect Misattribution Procedure.

Upon their arrival at the lab, participants met the experimenter and the two confederates (which were introduced as participants). It should be noted that in our previous study^42^, participants never met the confederates in person. As we will propose in the Discussion, this is an important difference that might have, in part, influenced our results. The three participants (one real, two confederates) were then told that they were about to play an interactive computer-based game in different rooms followed by a motor interaction task. After this explanation, we acquired their pictures for the time estimation task, then the participant was moved to a separated room to perform the cooperative time estimation task alone. The experiment started with a first AMP block that served as a baseline measure of implicit preference toward the two confederates. Immediately after, subjects underwent the status-inducing procedure (i.e., an interactive time estimation task), followed by a second AMP block. This second block was intended to measure whether the competence-based hierarchy established during the game changed the implicit preference toward the two confederates in terms of perceiving one of them as high-status (preferred) and the other as low-status (not preferred). The first confederate was then brought back in the participant′s room and the pair was engaged in the joint grasping task while their behaviour and motion kinematics was measured. Participants performed the task with Confederate 1 and then with Confederate 2 (order and confederate-status combination counterbalanced across participants). Finally, the participant was left again alone in the room and the last AMP block was administered to measure any possible change in implicit interpersonal preference due to the motor interaction itself (see Fig. 3 for a schematic representation of the events). At the end of the experiment, participants underwent a funnel debriefing procedure^46,47^ to determine if they had any suspicion about the cover story and were then debriefed (Fig. 3).

### Data handling

For the Joint Grasping task, we collected the following behavioural measures:

1. Asynchrony (absolute time delay between Subject’s and Confederate’s grasping time);
2. Start Delay (difference between Subject’s and Confederate’s start movement time).

For all behavioural and motion kinematics indexes, as a first step, data were cleaned by removing (1) erroneous trials (i.e., trials in which the pairs failed to accomplish the opposite/same or power/precision instruction), and (2) trials in which Asynchrony or StartDelay was higher than 2.5 standard deviations above the mean or smaller than 2.5 standard deviation below the mean. By these criteria, we removed 15% of trials for Asynchrony and 13% of trials for Start Delay.

### Statistical analyses

Data from the AMP and the Joint Grasping tasks were analysed with Multilevel Linear Mixed Models using the software R and the package lme4 (version 1.1-21)^48^. For the random structure, we aimed at including the by-participants random slopes and intercepts for each fixed effect. However, in some cases this approach led to a failure in model convergence and one or two fixed effects were removed from the random structure. Statistical significance of fixed effects was determined using type III ANOVA test with the *mixed* function from *afex* package^49^. Post-hoc comparisons and estimates of slopes of the covariate trend for each level of the factor were performed with the ‘Estimated Marginal Means’^50^ R package (version 1.3.3) via the *emmeans* and *emtrends* functions, respectively, and Tukey correction for multiple comparisons. The *emtrends* function allows to assess the statistical significance of simple slopes (i.e., if a slope is significantly different from zero) and of simple slopes differences (i.e., if the slopes are significantly different from each other), thus allowing testing for interactions between categorical factors and continuous variables.

The AMP mixed model included *Status* (High, Low) and *Session* (1, 2, 3) and their interactions as fixed factors. Because of convergence issues with more complex models, the random part included the by-participants intercept. The full structure of the AMP model in R notation was:

- AMP index ~ SESSION * STATUS (1 |Subject)

The Joint Grasping Task models for behavioural and kinematics data included as a continuous predictor an index of the effect of the status-inducing procedure on implicit preference toward the two confederates. We reasoned that any status effect on the Joint Grasping task might have been mediated by the effect of the procedure on implicit liking (AMP). Namely, some participants could have been more susceptible to the AMP procedure than others. The continuous predictor was extracted as follows: We first created two Session-effect indexes from the raw AMP values [HS-effect = (HS Session 2) − (HS Session 1), and LS-effect = (LS Session 2) − (LS Session 1)], then we created a Status effect index (HS-effect − LS-effect) which we called “Preference”. Positive values of the Preference index indicate a preference for the high-status partner while negative values indicate a preference for the low-status partner. The AMP status effect index (Preference) was then entered as a continuous predictor in our models for the Joint Action task analyses.

The Asynchrony and Start Delay models included, *Task* (Interactive, Cued), *Trial* (Opposite, Same), *Status* (High, Low), *Preference* (i.e., Status effect index of the AMP task) and their interactions as fixed factors. For the Asynchrony model, the random structure included the intercept and the random slope by participants for *Task*, *Trial* and *Status*. Because of convergence issues, for the StartDelay model (model 2), the random structure included the intercept and the random slope by participants for *Task* and *Status.* The full structure of the models in R notation is:

1. Asynchrony ~ TASK * TRIAL * STATUS * (Preference) + (TASK + TRIAL + STATUS | Subject)
2. StartDelay ~ TASK * TRIAL * STATUS * (Preference) + (TASK + STATUS |Subject)

Explicit ratings of Competence, Intelligence, Dominance and Attractiveness for the High and low status confederates were compared using paired-sample t tests with the *t.test* function from the package Stats (version 3.6.0). Analyses on motion kinematics indexes are reported in Supplementary Information.

## Results

### Affect misattribution procedure (AMP)

**Figure 4:**
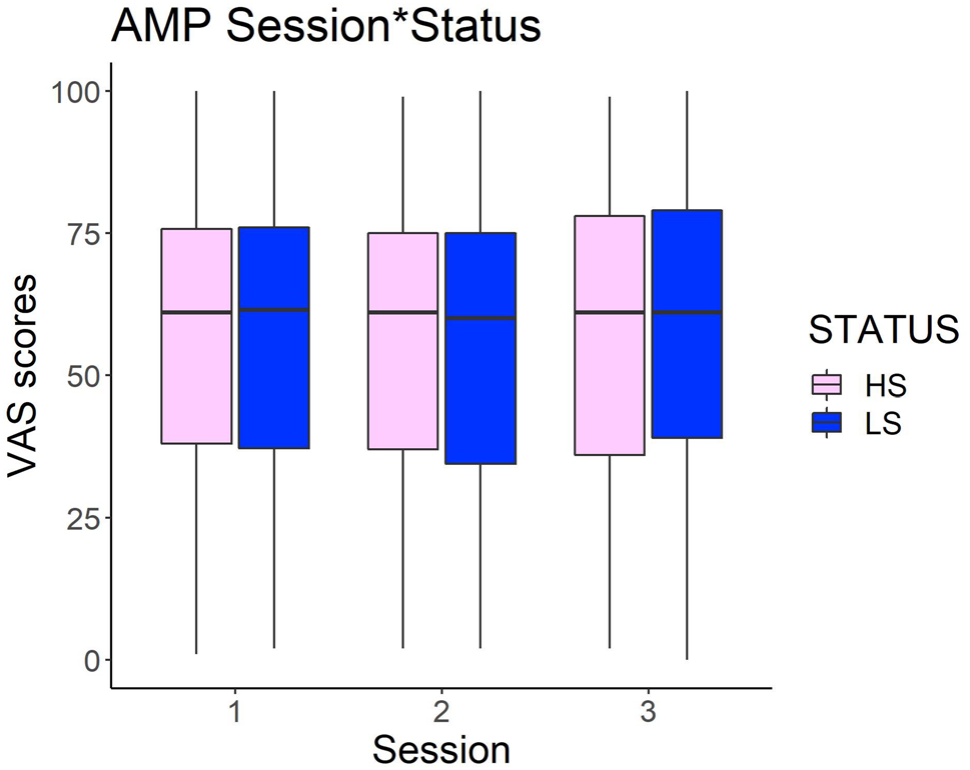
Vas scores attributed to the neutral primes preceded by the high status (HS) and the low status (LS) confederates’ picture in Session 1 (before the status manipulation), Session 2 (after the status manipulation) and Session 3 (after the Joint Grasping task). Horizontal lines in the boxes indicate the median, upper and lower borders indicate 1st and 3rd quartile, "whiskers" extend to the farthest points that are not outliers.

Type 3 ANOVA on VAS scores revealed that both the Status (*F*(1,2029) = 0.35, *p* = 0.55) and the Session (*F*(1,2029) = 0.87, *p* = 0.41) factors were nonsignificant. The Status*Session interaction was also nonsignificant (*F*(1,2029) = 0.20, *p* = 0.81). This suggests that participants’ implicit preference for the two confederates (1) did not change through the three sessions and (2) was not modulated by the confederates’ status (see Fig. 4). We decided to further explore this result by carrying out additional analyses only on the first two sessions (i.e., before and after the status-inducing procedure) in an attempt to replicate the results of our previous experiment^42^ on which basis we expected the pleasantness ratings of target pictures associated with the high status to be higher than those associated with the low status in Session 2 (i.e., after the status-inducing procedure). We created a new model with only two levels for the Session factor (i.e., Session 1 and Session 2). Again, type 3 ANOVA failed to show any significant main effect or interaction (all Fs < 1, all ps > 0.1), that is, these results did not conform to expectations, based on Boukarras et al.^42^.

#### Explicit ratings

**Figure 5:**
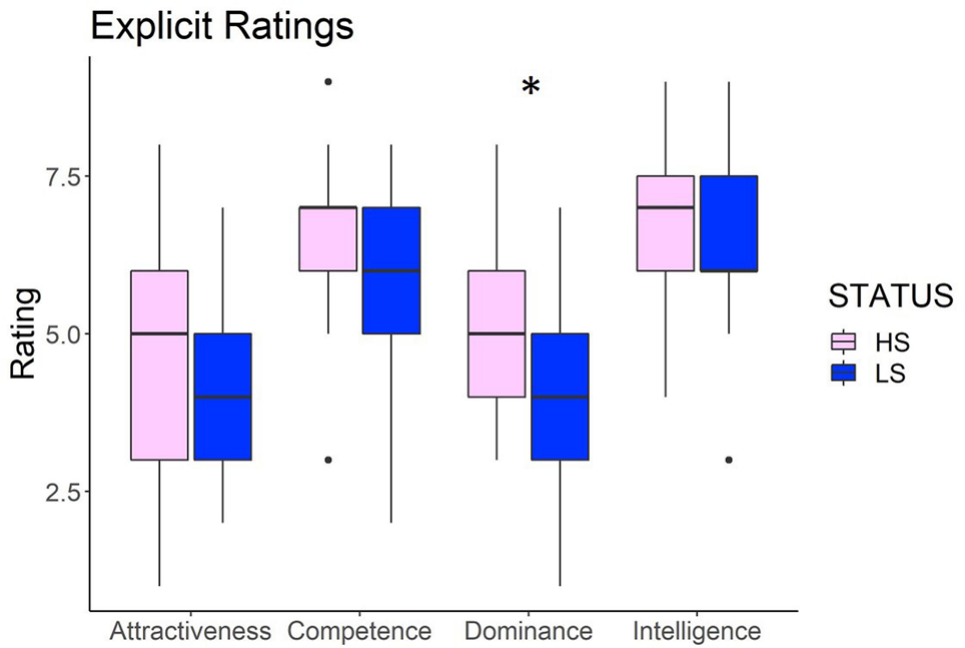
Explicit ratings of the High and Low status confederate on Attractiveness, Competence, Intelligence and Dominance. Asterisks indicate *p* values: *p* < 0.05. Horizontal lines in the boxes indicate the median, upper and lower borders indicate 1st and 3rd quartile, "whiskers" extend to the farthest points that are not outliers, dots represent outlier trials.

Results from paired-samples t-tests showed that after the status-inducing procedure, participants rated the high-status confederate as more Dominant than the low status confederate (*t* (18) = 2.17, *p* = 0.04). We found no significant difference in the ratings of Competence (*t*(18) = 1.05, *p* = 0.30), Intelligence (*t*(18) = 0.56, *p* = 0.57) and Attractiveness (*t*(18) = 1.35, *p* = 0.19), see Fig. 5.

### Joint grasping task

#### Asynchrony

**Figure 6:**
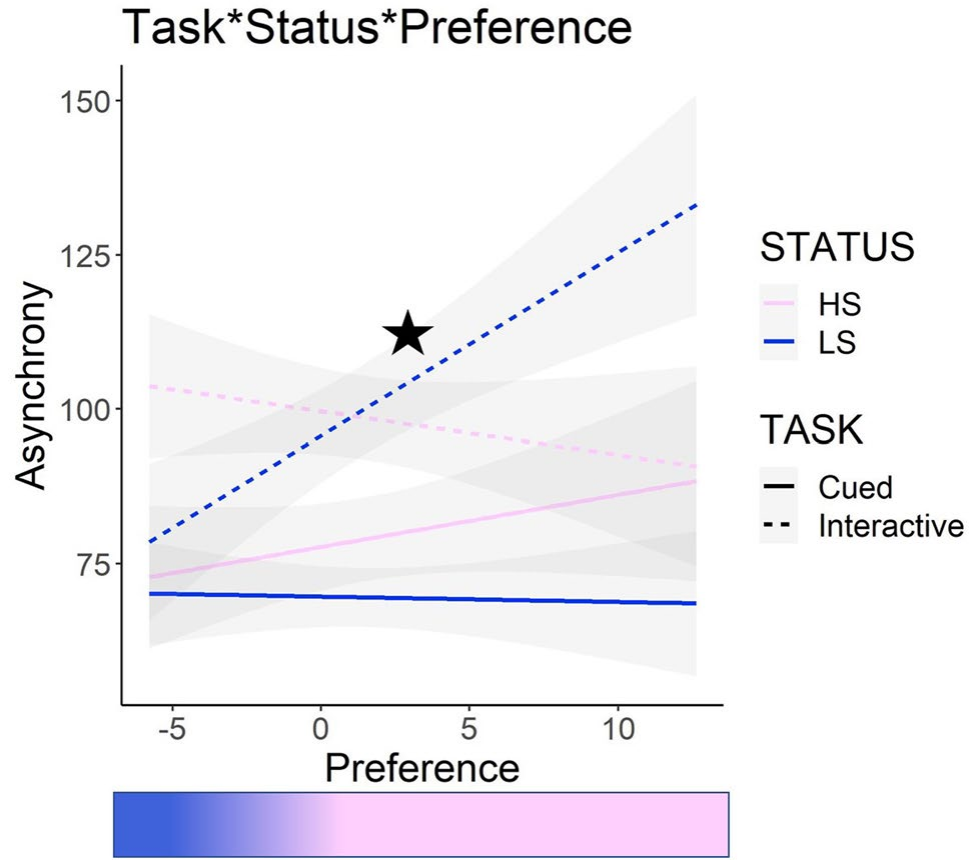
Grasping Asynchrony with the high- and low-status confederates during the Interactive task was modulated by participants’ implicit preference. Positive values of Preference (right side of the x axis) indicate a preference for the high-status, while negative (left side of the x axis) values indicate a preference for the low-status. Smaller values of Asynchrony indicate a better performance. A star indicates a simple slope that is significantly different from zero (i.e., the 95% confidence interval does not contain zero).

Type 3 ANOVA revealed a significant main effect of Task (*F*(1,15.68) = 16.78, *p* < 0.001), indicating that Asynchrony was reduced in the Cued task, compared to the Interactive one. Moreover, we found a significant Task*Status*Preference interaction (*F*(1,3578) = 22.02, *p* < 0.0001). As a follow-up test on interactions with the continuous predictor Preference, we used the R function *emtrends* to estimate the *slopes* of the covariate trend for each level of the factors. This analysis revealed that the slope of Interactive low-status as a function of Preference values was significantly different from zero (LCL = 0.34, UCL = 5.84)—that is, only when interacting with the low-status partner did a preference for him (negative Preference values) allow better synchrony during the Interactive task. During the Interactive block with the low-status confederate, Asynchrony yielded an increase of 3.09 ms for each 1-unit increase in Preference (i.e., indexing a preference for the high-status confederate) (SE = 1.40). Thus, the stronger the implicit preference was for the low- over the high-status participant after the status manipulation (i.e., negative Preference values), the smaller was the Asynchrony when interacting with the low-status. The pairwise difference between the simple slopes of high- and low-status in the Interactive task was not significant (estimate =  − 3.90, SE = 1.72, z-ratio =  − 2.26, *p* = 0.10), nor it was the simple slope of Interactive_high-status. However, although not significantly, during the Interactive block with the high-status confederate, Asynchrony yielded a *decrease* of 0.81 ms for each 1-unit increase in Preference (SE = 1.97), thus suggesting that the stronger the implicit preference was for the high- over the low-status after the status manipulation (i.e., positive Preference values), the *smaller* the Asynchrony when coordinating with the high-status in the Interactive condition (see Fig. 6).

#### Start delay (RTs)

Type 3 ANOVA revealed a significant main effect of Trial (*F*(1,3623) = 8.04, *p* = 0.005), indicating that participants started their movements earlier with respect to the confederate during Imitative, compared to Complementary trials and a Task*Status interaction (*F*(1,3623) = 7.36, *p* = 0.007). Post-hoc tests showed no significant difference between the High and Low status conditions in the Cued block (estimate = 2.22, SE = 15.3, z-ratio = 0.14, *p* = 0.884) nor in the Interactive one (estimate = 17.42, SE = 15.3, z-ratio = 1.14, *p* = 0.254). To further investigate the significant interaction, we first ran one-sample pairwise t-tests against zero to check which conditions were significantly different from zero. StartDelay values were significantly different from zero in the Interactive condition for both the High (*p* < 0.0001) and the Low (*p* < 0.0001) status conditions but the same test was nonsignificant for the Cued task (all Ps > 0.857). We then ran one-sided paired sample t tests (alternative “greater”) to compare StartDelay values in the Interactive block with values in the Cued block, separately for the High and Low status conditions. For the High status condition, StartDelay was longer in the Interactive compared to the Cued condition (t(1,17) = 1.86, *p* = 0.039), while the same comparison was marginally significant for the Low status condition (t(1,17) = 1.60, *p* = 0.063). Although this might explain the significant Task x Status interaction, our results indicate that the social status of the interaction partner did not affect StartDelay.

## Discussion

Previous studies investigating how status shapes motor processes have relied on action observation-execution tasks^30^ or on the Joint Simon paradigm^29^ in which two participants act independently while observing or taking into account a partner’s action—importantly, without the need to coordinate with them. The Joint Grasping paradigm used in the present study closely mimics a real-life motor interaction setting, where the partner’s action, rather than being ignored or incidental, is necessarily included in one’s own motor plan to achieve the task goal^51^. Our results suggest that the social status of the interaction partner modulates realistic motor interactions.

Participants’ ability to synchronise their reach-to-grasp movements with those of the interaction partner varied as a function of their implicit preference for him only when interacting with the low status confederate. Specifically, we observed that grasping asynchrony with the low-status partner decreased as participants’ implicit preference for him increased. This result was specific for the Interactive task, being absent in our control (Cued) task. The interactive task used in the present study preserves the features of realistic motor interactions, requiring both the temporal and spatial coordination of the two partners’ movements and the integration of one’s own movements with predictions about the others’ actions^12^. Our results suggest that this ability to integrate observed and executed movements into a smooth motor plan^51^ is modulated by implicit attitudes toward the interaction partner. This result is reminiscent of observations in a previous study^12^, in which inducing a negative interpersonal relationship into pairs of participants decreased the tendency to mutually adapt—a marker of human–human joint-actions^34,51,52^ during the same joint grasping task. Thus, while results from previous studies support the idea that the actions performed by high-social-status models are co-represented more than those performed by someone of low status^29^, the present data seem to show that preference, rather than individuals’ status per se, may guide the ability to simulate and predict the actions of a partner. However, in our study, task performance when interacting with the high-status confederate was not influenced by implicit preference. This suggests that participants’ ability to simulate and predict the actions of high-status individuals was somehow impervious to their attitude towards him.

Results from the Start Delay index suggest that participants started their movements later with respect to the confederate in the Interactive but not in the Cued task. However, no significant difference was observed when directly comparing Start Delay in the High and Low status conditions, thus indicating that the social status of the interaction partner did not affect whether participants would start their movements before of after the confederate.

Based on the findings reported in our previous study^42^, we expected that, after the time estimation game, participants would give higher ratings of pleasantness to the neutral targets preceded by the high status prime than to those preceded by the low status prime. Contrary to our expectations, the implicit preference for the high-status player was not significantly different from that of the low status after the status-inducing procedure (AMP session 2) nor after the joint grasping task (AMP session 3). This seeming discrepancy with our previous study^42^ may reflect a methodological difference. In the present study, participants actually met the two confederates before starting the status-inducing procedure, while in the previous study participants never met the partners (see Methods section). As a result, during their first encounter, participants might have formed an impression of the two confederates and developed an implicit preference for one of the two. This preference, based on a first impression, might have then become (at least in some participants) impermeable to the status-inducing procedure. Indeed, research in social psychology seems to suggest that, while explicit impressions can be corrected on the light of new information, implicit first impressions are harder to change^53^, unless the new information is highly negatively diagnostic^54^. It is therefore possible that our status-inducing procedure was not strong enough to counteract the first-sight implicit impression that participants formed of the two confederates. It follows that implicit preferences toward the two confederates might have been influenced by both the first impression *and* the acquired status, therefore leading to higher levels of variability. This would explain why, while a direct comparison between the preference ratings before and after the status inducing procedure did not disclose any difference between high- and low-status partners, inserting the preference ratings as covariate in the analyses of the interaction task showed a role of preference.

Our results show that the only dimension in which the explicit rating of the two confederates was influenced by their social status was Dominance. Although some scholars^15,55^ have argued that competence and dominance are two distinct pathways for status acquisition, some others have raised the proposal that dominance itself requires high levels of competence in specific domains, such as handling weapons, recruiting allies or being socially manipulative^56^. Surprisingly, we found that explicit ratings of Competence and Intelligence for the high-status confederate were not significantly different from those given to the low-status one. As for the implicit ratings, meeting the two confederates before the game, and shaking their hands^57^ might have influenced participants’ explicit evaluation. Another possibility is that the sample size was not adequate to capture the effect of status on both implicit and explicit measures. Our power calculation was based on an effect size derived from a motor interaction experiment with a similar manipulation^11^. This effect size was comparable to a Cohen’s d of 1.6 and, thus, rather large for a psychological science study. Therefore, it is possible that while our study had enough power to detect an effect in the Joint Grasping Task, it was underpowered for the implicit and explicit measures. Indeed, in our previous study^42^ the sample size was slightly larger (N = 26) than in the present one, so the lack of power might explain the discrepancies between the two.

The present study only tested male participants interacting with male confederates. However, gender-based differences have been observed in the susceptibility to status inductions, with larger effects on male participants when status is competition-based^25^ and larger effects on female participants with occupational status^58^. This suggests that men and women might be sensitive to different aspects of social status. Although we stressed the cooperative nature of the time estimation task, it possible that our (male) participants experienced the game as a competition. For this reason, the observed effects might be due to a gender-related specific sensitiveness to competition-based status. Future research could examine the interactions between gender and specific status determinants, such as occupational or economic status, on the dynamics of motor interactions.

## Supplementary Information

Supplementary Information 1.

Supplementary Information 2.

Supplementary Information 3.

## Data Availability

The datasets generated and/or analysed during the current study are available in the Open Science Framework (OSF) repository, https://osf.io/m4k7a/?view_only=310bf0d44de14c64ba92458fc4ee70e8. The experiment was not preregistered.
