# Supplementary material for: Competence-based social status and implicit preference modulate the ability to coordinate during a joint grasping task
Source: Sci Rep. 2021 Mar 5;11:5321. doi: 10.1038/s41598-021-84280-z (PMC7935999; doi:10.1038/s41598-021-84280-z)
Supplement: Supplementary file 1 — Supplementary Information 1. [file 41598_2021_84280_MOESM1_ESM.docx]

**Competence-based social status and implicit preference modulate the ability to coordinate during a joint grasping task**

Boukarras^1,2*^, Era^1,2^, Aglioti^2,3^, Candidi^1,2*^

1. Department of Psychology, Sapienza University, Rome, Italy
2. IRCCS Fondazione Santa Lucia, Rome, Italy
3. Sapienza University of Rome and CNLS@Sapienza Istituto Italiano di Tecnologia, Genova

# Supplementary information

**Supplementary Methods**

*Affect Misattribution Procedure (AMP)*

The AMP task was delivered using E Prime 2.0 (Psychology Software Tools, Pittsburgh, PA). Each trial started with a fixation cross presented for 1000 ms at the centre of the screen, followed by the prime image (identity of either Confederate 1 or 2) for 75 ms, a backward mask for 100 ms and the target (ideogram) for 1000 ms (as in 40 and 45). Following each target (and backward mask), a vertical Visual Analogue Scale (VAS, height 10 cm) appeared on the screen under the sentence “How much do you like this image?”, with the words “Not at all” and “Extremely” beside its bottom and top end, respectively, and lasted on the screen until a response was made.

*Status-inducing procedure*

The task was administered with E-Prime 2.0 (Psychology Software Tools, Pittsburgh, PA). To make the task more engaging and avoid ceiling effects, we used a staircase procedure. At the beginning of the task, the allowable response time was set at 1 second +/- 550 ms (i.e., a response was scored as correct if falling between 550 and 1550 ms after the moment in which the blue circle turned green). If the participant response fell within the allowable response time, his performance was scored 5, and the threshold was set at 1 second +/- 500 ms. Otherwise, the threshold was incremented at 1 second +/- 600 ms and the player’s response fell outside the allowable response time. The task consisted of 8 blocks. In the first four blocks we kept the hierarchy unstable, with the participant moving through the first (block 1), second (block 2) and third (blocks 3 and 4) positions. From block 5 to 8, however, the ranking remained stable, with the experimental subject in the middle position. Whilst the participant’s displayed score reflected his real performance, we covertly manipulated the scores obtained by the two fake players so that the one who was to become the “high-status” ranked first from block 5 to 8, and the “low-status” ranked last in the same 4 blocks. At the end of the task we presented a slide displaying the final hierarchy and the collective score.

*Joint Grasping Task*

Each participant-confederate dyad was seated at the opposite sides of a small table (120 x 100 cm) and was instructed to reach and grasp a bottle-shaped object placed in front of them (40 cm away from the participant and 5 cm from the midline) following auditory instructions delivered via headphones. Both the participant and the confederate had their own bottle-shaped object, which was constituted by two superimposed cylinders with different diameters (small, 2.5; large, 7.0 cm). Given their shape, the upper cylinder was to be grasped with a precision grip (index finger and thumb) while the lower cylinder was to be grasped with a power grip (whole hand). Before the start of each trial, participants were asked to keep pressed between their right index and thumb fingers a starting button placed 40 cm away from the bottle-shaped object and 10 cm to right of the midline. Start movement time was recorded from button release, while touch-time on the bottle was recorded via two pairs of touch-sensitive copper plates that were placed on each cylinder at 15 cm and 23 cm of the total height of the object.

Each participant had three infrared reflective markers (5 mm diameter) each attached to: i) thumb, ulnar side of the nail, ii) index ﬁnger, radial side of the nail and iii) wrist, dorso-distal aspect of the radial styloid process. Movement kinematics were tracked and recorded with a SMART-D motion capture system (Bioengineering Technology & Systems [B|T|S]). Four infrared cameras with wide-angle lenses (sampling rate 100 Hz) were placed about 100 cm away from each of the four corners of the table captured the movements of the markers in 3D space. Auditory instructions concerning the movement to be executed were delivered simultaneously to both participants via headphones. The instructions took the form of four different sounds corresponding to two different experimental conditions (see below): i) a ‘‘high-pitch’’ (1479 Hz), ii) a ‘‘low-pitch’’ (115.5 Hz), iii) a voice saying ‘‘opposite”, iv) a voice saying “same”.

## Supplementary Results on kinematic parameters (hand aperture and wrist height)

As motion kinematics measures, we collected (only for the experimental subject):

1. Max grip Aperture (MaxAp): index-thumb maximum grip aperture (maximum 3-D Euclidean distance);
2. Max Wrist Height (MaxH): maximum height reached by the wrist along the reaching phase.

Data were cleaned by removing 1) erroneous trials (i.e., trials in which the pairs failed to accomplish the opposite/same or power/precision instruction), and 2) trials in which MaxAp or MaxH was higher than 2.5 standard deviations above the mean or smaller than 2.5 standard deviation below the mean. By these criteria, we removed 14% of trials for MaxAp and 14% of trials for MaxH.

The Max Aperture and Max Wrist Height models included *Task* (Interactive, Cued), *Trial* (Opposite, Same), *Movement* (Power, Precision), *Status* (High, Low), *Preference* (i.e., Status effect index based on the score of the AMP task) and their interactions as fixed factors. The random part included the intercept and the random slope by participants for *Task*, *Trial, Movement* and *Status*. The full models in R notation are:

- Max Aperture ~ TASK * TRIAL * MOVEMENT*STATUS * Preference + (TASK + TRIAL+ MOVEMENT+STATUS | Subject)
- Max Wrist Height ~ TASK * TRIAL * MOVEMENT*STATUS * Preference + (TASK + TRIAL+ MOVEMENT+STATUS | Subject)

### Max grip aperture

Type 3 ANOVA revealed a significant main effects of Movement (*F*(1,16.07) = 255, *p* < 0.001). As expected, the maximum peak of grip aperture was larger for power than for precision grips. A significant Trial*Movement interaction (*F*(1,3640) = 5.59, *p* = 0.01) revealed that grip aperture for precision grips was larger during Complementary, compared to Imitative, trials (estimate = 1.36, SE = 0.44, z-ratio = 3.10, p = 0.001). This result indicates the emergence of visuo-motor interference, as participants were likely imitating the hand posture of the interaction partner during complementary actions (see Fig. S1). There was also a significant Task*Movement interaction (*F*(1,3564) = 87.84, *p* < 0.001). Post-hoc tests, however, indicated that Max Aperture was larger for power than for precision grips both in the Cued (estimate = 29.41, SE = 1.70, z-ratio = 17.34, *p* < 0.0001) and in the Interactive (estimate = 23.95, SE = 1.70, z-ratio = 14.12, *p* < 0.0001) tasks. Post hoc tests also showed that grip aperture for precision grips was reduced in the Cued, compared to the Interactive task (estimate = -5.17, SE = 1.69, z-ratio = -3.06, p = 0.01). Type 3 ANOVA also revealed significant Task*Status (*F*(1,3625) = 19.02, *p* < 0.001) and Status* Movement *Preference (*F*(1,3648) = 14.06, *p* < 0.001) interactions. However, follow-up tests failed to show any significant result (all Ps > 0.35).

INSERT FIGURE S1

### Max wrist height

Type 3 ANOVA revealed significant main effects of Trial (*F*(1,15)= 12.32, *p* = 0.003) (*F*(1,15)= 10.24, *p* = 0.006) and Movement (*F*(1,16)= 1316, *p* < 0.001). Max Wrist Height was higher for Opposite compared to Same trials and, as expected, for Precision compared to Power grasping. We also found a significant Task*Trial*Status*Movement*Preference interaction (*F*(1,3630) = 5.71, *p* = 0.017). Simple slope analysis performed with the function *emtrends* revealed that, only for the Interactive task, the slopes of High-status Opposite Power, Low-status Opposite Power, Low-status Same Power, High-status Opposite Precision, Low-status Opposite Precision, High-status Same Precision and Low-status Same Precision were all significantly different from zero. Simple slopes comparison revealed a significant difference only in the Interactive_power condition between the slopes of High-status_Opposite and High-status_Same (estimate = - 1.15, SE = 0.28, z-ratio = -4.04, *p* = 0.001) while the same comparison was not significant for Low-status. This indicates that increasing Preference for the high-status partner has the effect of decreasing Max Wrist Height during Power-Opposite trials while slightly increasing it in Power-Same Interactive trials during the interaction with higher status. Indeed, when participants were interacting with the High-status confederate, visuo-motor interference was dependent on their Preference level (see Figure S2). Surprisingly, the less participants preferred the high- to the low-status partner, the higher was their visuo-motor interference with the high-status confederate (i.e., a larger difference between Opposite-Power and Same-Power).

INSERT FIGURE S2

## Supplementary discussion

### Emergence of visuo-motor interference during complementary trials (Max Grip Aperture)

Participants’ hand aperture for precision grasping increased with Complementary, with respect to Imitative, trials. Since in this condition participants were asked to perform precision grasping while observing a power one, this effect is likely due to a form of visuo-motor interference, whereby the observation of an incongruent movement influences the pre-shaping of an ongoing grasping movement. This evidence is in line with previous results from studies employing the Joint Grasping Task [1,2] and supports the view that sensorimotor simulation processes, most likely involving the so-called “mirror neuron system”, are recruited during the execution of joint actions.

### Implicit preference decreases visuo-motor interference (Max Wrist Height).

Our results on wrist height showed an inverse relationship between visuo-motor interference and implicit preference for the high-status confederate. Participants’ automatic imitation of the high-status’ movements during complementary trials (as indexed by an increase in wrist height with respect to imitative trials) increased as their implicit preference for the high status decreased. Although previous research has shown increased involuntary imitation toward in-group models [3,4], therefore suggesting that we might be more likely to imitate those whom we like more [5], the results from a study that used our same task seem to suggest a different picture. Sacheli and colleagues [2] tested pairs of participants that did or did not receive a negative interpersonal manipulation and found visuo-motor interference effects in the negatively manipulated but not in the neutral group. The authors proposed that the presence of visuo-motor interference could be accounted for by the fact that the negative interpersonal relationship might have prevented a smooth integration of the partners’ motor plans. The results of the current study show a conceptually coherent pattern, in which visuo-motor interference for the high-status’ observed actions increases as the preference for him decreases. A recent study from Sacheli and colleagues [6] found that performing complementary interactions is not more difficult than performing imitative ones in a joint action task. The authors proposed that, during joint action participants, rather than passively simulating the interactor’s movements, recruit predictive processes thanks to which movements by others can be integrated the into a Dyadic Motor Plan [6]. Our results suggest that the degree to which one’s own and others’ motor plans are integrated into a shared motor representation could be dependent on motivational and social factors. In this vein, interacting with a non-preferred high-status partner might have turned the joint action into “acting while observing another”. Thus, automatic imitation of complementary movements implies that when interacting with a disliked partner, it is not easy to “move together” with him and achieve a shared goal. In this type of circumstance, it may be the case that participants move “while observing another movement” and therefore display interference effects [6,7]. The interaction between implicit preference and motor interference was only observed in the Interactive task and not in the Cued one. This might be explained by the fact that during the Interactive task participants were required to continuously monitor what the partner is doing, thus leaving more room for interference effects to arise.

**Supplementary references**

1. Era, V., Aglioti, S. M., Mancusi, C., & Candidi, M. (2020). Visuo-motor interference with a virtual partner is equally present in cooperative and competitive interactions. Psychological research, 84, 810-822.
2. Sacheli, L. M., Candidi, M., Pavone, E. F., Tidoni, E., & Aglioti, S. M. (2012). And yet they act together: interpersonal perception modulates visuo-motor interference and mutual adjustments during a joint-grasping task. *PloS one*, *7*(11), e50223.
3. Bourgeois, P., & Hess, U. (2008). The impact of social context on mimicry. *Biological psychology*, *77*(3), 343-352.
4. Sacheli, L. M., Christensen, A., Giese, M. A., Taubert, N., Pavone, E. F., Aglioti, S. M., & Candidi, M. (2015). Prejudiced interactions: implicit racial bias reduces predictive simulation during joint action with an out-group avatar. *Scientific Reports*, *5*, 8507.
5. Miles, L. K., Griffiths, J. L., Richardson, M. J., & Macrae, C. N. (2010). Too late to coordinate: Contextual influences on behavioral synchrony. *European Journal of Social Psychology*, *40*(1), 52-60.
6. Sacheli, L. M., Arcangeli, E., & Paulesu, E. (2018). Evidence for a dyadic motor plan in joint action. *Scientific reports*, *8*(1), 5027.
7. Kilner, J. M., Paulignan, Y., & Blakemore, S. J. (2003). An interference effect of observed biological movement on action. *Current biology*, *13*(6), 522-525.

SUPPLEMENTARY FIGURES CAPTIONS

Fig. S1 – Visuo-motor interference effect. Max Grip aperture for precision movements was larger during complementary (opposite) than imitative (same) trials. Asterisks indicate p values: *p* < 0.05 (*), *p* < 0.01 (**), p < 0.001 (***). Horizontal lines in the boxes indicate the median, upper and lower borders indicate 1^st^ and 3^rd^ quartile, "whiskers" extend to the farthest points that are not outliers, dots represent outlier trials.

Fig S2 – Status*Task*Trial*Movement*Preference interaction. When interacting with the low status confederate, participants’ wrist height was higher for Opposite than for Same Power grasping. This suggests that participants were involuntarily imitating the low-status confederate’s movement (i.e., a Precision grasping). When interacting with the high-status confederate, participants’ visuo-motor interference (i.e., higher wrist height for Opposite than for same Power grasping) was dependent on their implicit evaluation of the partner. Asterisks indicate p values: *p* < 0.05 (*), *p* < 0.01 (**), p < 0.001 (***). Stars indicate simple slopes that are significantly different from zero (i.e. the 95% confidence interval does not contain zero).
