# Supplementary figures and images for: Competence-based social status and implicit preference modulate the ability to coordinate during a joint grasping task
Source: Sci Rep. 2021 Mar 5;11:5321. doi: 10.1038/s41598-021-84280-z (PMC7935999; doi:10.1038/s41598-021-84280-z)

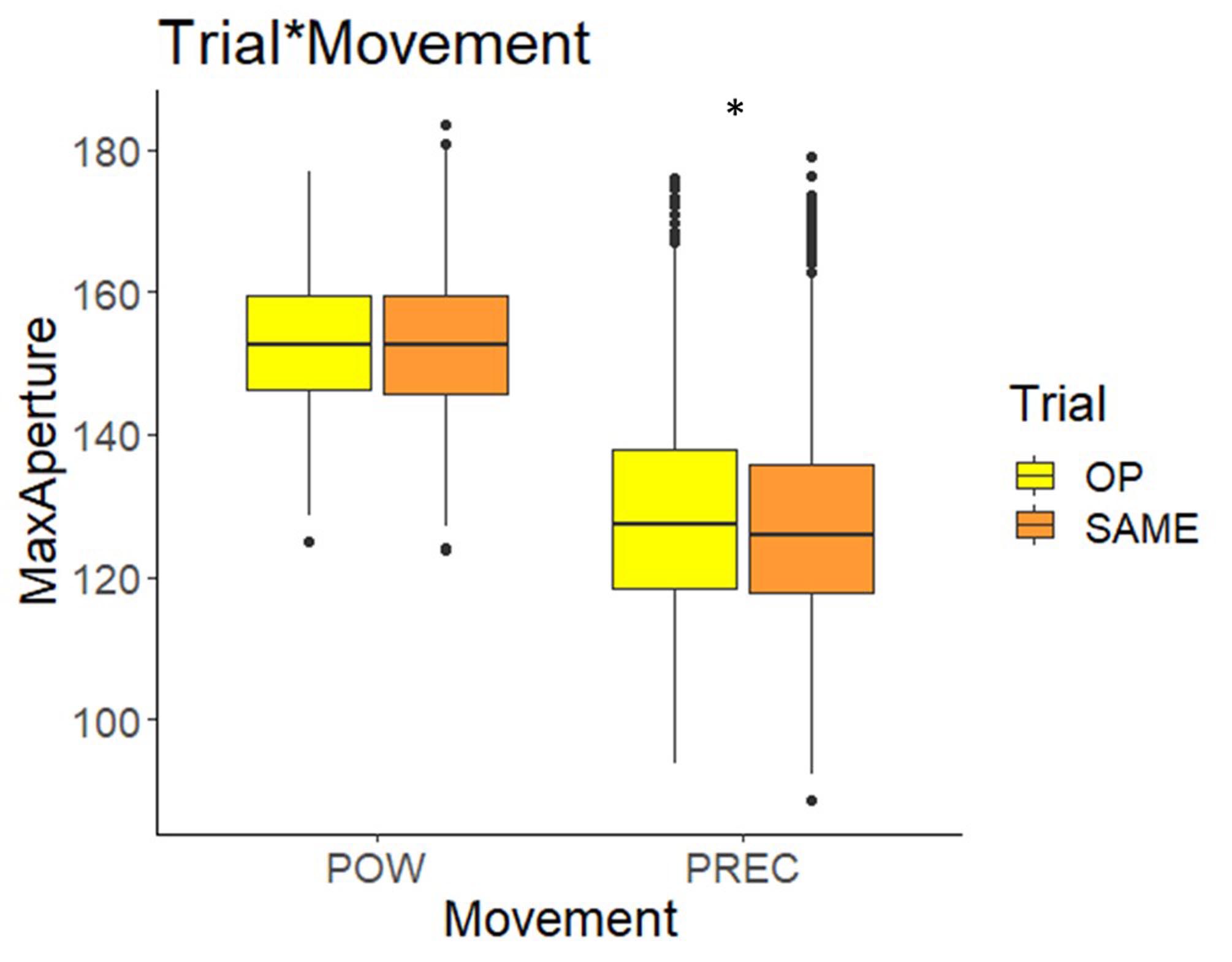

Supplement: Supplementary file 2 — Supplementary Information 2. [file 41598_2021_84280_MOESM2_ESM.jpg]

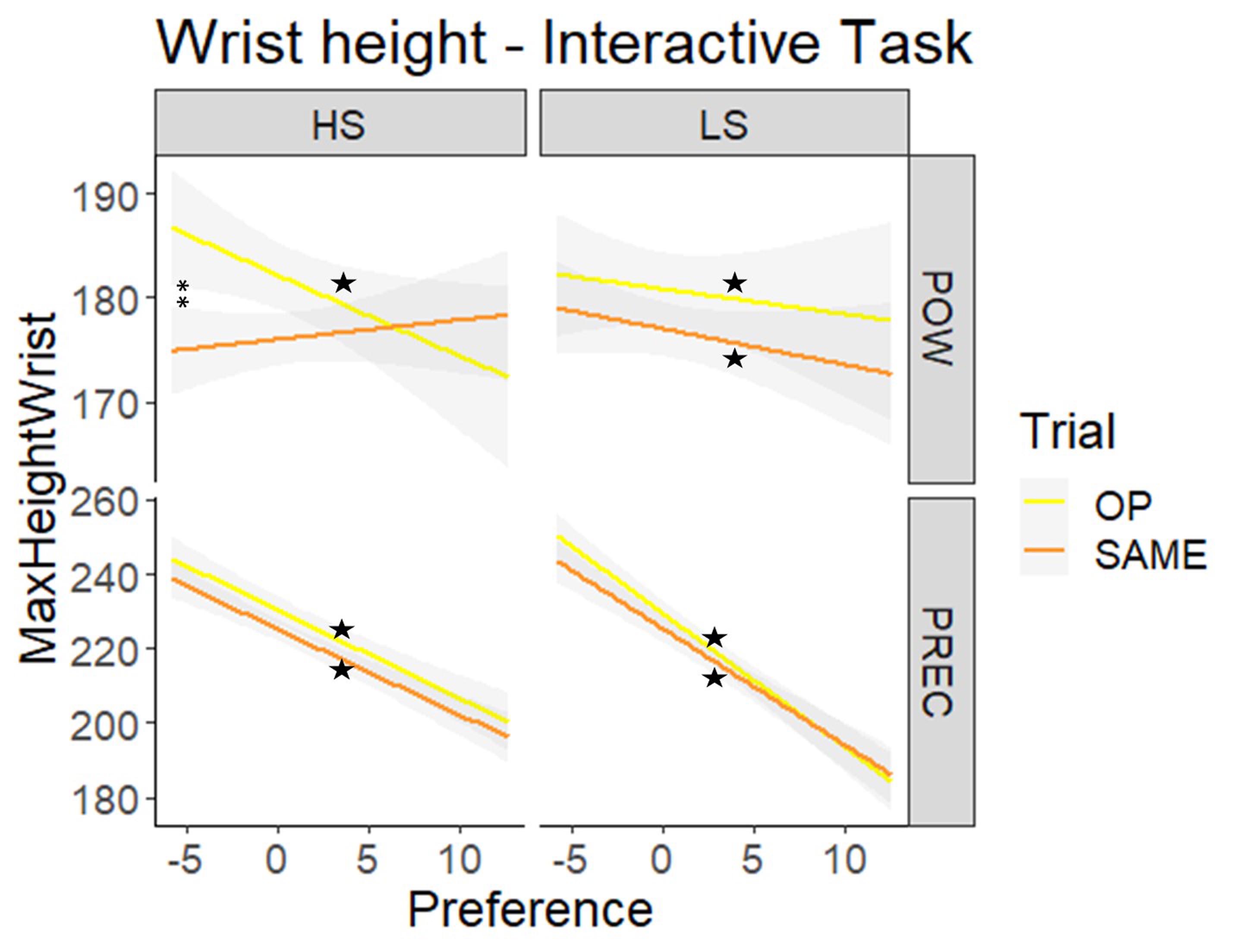

Supplement: Supplementary file 3 — Supplementary Information 3. [file 41598_2021_84280_MOESM3_ESM.jpg]
